# Resistance gene pool to co-trimoxazole in non-susceptible *Nocardia* strains

**DOI:** 10.3389/fmicb.2015.00376

**Published:** 2015-04-28

**Authors:** Sylvia Valdezate, Noelia Garrido, Gema Carrasco, Pilar Villalón, María J. Medina-Pascual, Juan A. Saéz-Nieto

**Affiliations:** Servicio de Bacteriología and Taxonomía, Centro Nacional de Microbiología, Instituto de Salud Carlos IIIMadrid, Spain

**Keywords:** *Nocardia* species, co-trimoxazole, antimicrobial resistant determinants, integrons

## Abstract

The soil-borne pathogen *Nocardia* sp. causes severe cutaneous, pulmonary, and central nervous system infections. Against them, co-trimoxazole (SXT) constitutes the mainstay of antimicrobial therapy. However, some *Nocardia* strains show resistance to SXT, but the underlying genetic basis is unknown. We investigated the presence of genetic resistance determinants and class 1–3 integrons in 76 SXT-resistant *Nocardia* strains by PCR and sequencing. By *E* test, these clinical strains showed SXT minimum inhibitory concentrations of ≥32:608 mg/L (ratio of 1:19 for trimethoprim: sulfamethoxazole). They belonged to 12 species, being the main representatives *Nocardia farcinica* (32%), followed by *N. flavorosea* (6.5%), *N. nova* (11.8%), *N. carnea* (10.5%), *N. transvalensis* (10.5%), and *Nocardia* sp. (6.5%). The prevalence of resistance genes in the SXT-resistant strains was as follows: *sul1* and *sul2* 93.4 and 78.9%, respectively, *dfrA*(*S1*) 14.7%, *bla*TEM-1 and *bla*Z 2.6 and 2.6%, respectively, *VIM*-2 1.3%, *aph*(*3*′)*-IIIa* 40.8%, *ermA*, *ermB*, *mefA*, and *msrD* 2.6, 77.6, 14.4, and 5.2%, respectively, and *tet*(O), *tet*(M), and *tet*(L) 48.6, 25.0, and 3.9%, respectively. Detected amino acid changes in GyrA were not related to fluoroquinolone resistance, but probably linked to species polymorphism. Class 1 and 3 integrons were found in 93.42 and 56.57% strains, respectively. Class 2 integrons and *sul3* genes were not detected. Other mechanisms, different than *dfr*A(*S1*), *dfr*D, *dfr*F, *dfr*G, and *dfr*K, could explain the strong trimethoprim resistance shown by the other 64 strains. For first time, resistance determinants commonly found in clinically important bacteria were detected in *Nocardia* sp. *sul1, sul2, erm*(B), and *tet*(O) were the most prevalent in the SXT-resistant strains. The similarity in their resistome could be due to a common genetic platform, in which these determinants are co-transferred.

## Introduction

*Nocardia* sp. are branching, aerobic actinomycetes found in soil and water, but which have an increasingly recognized role in human disease – a consequence of improvement in their isolation from immunocompromised patients. Indeed, these opportunistic bacteria mainly infect patients with deficient cell-mediated immunity (Minero et al., 2009; Ambrosioni et al., 2010; Welsh et al., 2013). Cutaneous infections are more prevalent in immunocompetent patients, while pulmonary and disseminated infections are more prevalent in the immunosuppressed patients (Brown-Elliott et al., 2006; Minero et al., 2009). Delays in diagnosis, a consequence of the absence of specific signs and symptoms, are associated with the progression to disseminated disease and recurrence. In such cases, prolonged antimicrobial treatment is required (Ambrosioni et al., 2010).

Traditionally, nearly all forms of nocardiosis have been treated with co-trimoxazole (trimethoprim/sulfamethoxazole or SXT), either alone or in combination with minocycline, amikacin, or β-lactams, depending on the organ involved, the severity of infection, and the presence of comorbidities (Ambrosioni et al., 2010; Welsh et al., 2013). SXT inhibits the enzymes involved in two consecutive steps of bacterial folic acid metabolic pathway, i.e., dihydropteroate synthetase (DHPS) and dihydrofolate reductase (DHFR; Huovinen, 2001).

**FIGURE 1:**
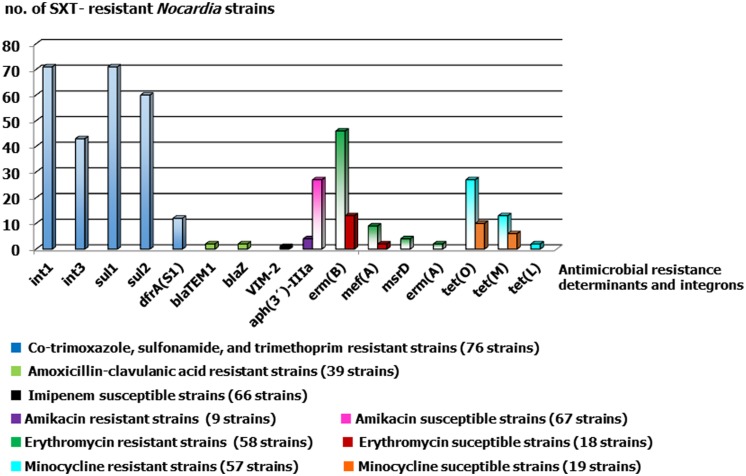
**Antimicrobial resistance determinants detected in SXT-resistant *Nocardia* strains considering their susceptibilities to the other studied antimicrobials**. The number of strains for each category and agent were showed between brackets.

The recognition of resistance to sulfonamide in *Nocardia* sp. is controversial (Deresinski, 2012). Great differences in the prevalence of sulfonamide resistance have been documented within countries, e.g., **Figure 1** of 42 and 2% have been reported from the USA. These discrepancies have been related to methodological differences and to difficulties in visually determining minimum inhibitory concentrations (MICs) by the broth microdilution (Uhde et al., 2010; Brown-Elliott et al., 2012). In our experience of identifying *Nocardia* submitted from across Spain, this type of resistance is seen in a reduced number of strains (less than 4%).

Descriptions of resistance determinants in *Nocardia* – β-lactamases – or possible determinants – second gyrase B, an extra copy of *rpo*B-, detected by partial or whole genome sequencing (Laurent et al., 1999; Poirel et al., 2001; Vera-Cabrera et al., 2013), led us to examine, in clinical strains strongly resistant to SXT, the diversity of acquired antimicrobial resistance determinants commonly detected in clinical and environmental bacteria and the presence of integrons as vehicles of the resistance gene recruitment.

## Materials and Methods

### Bacterial Strains and Identification

Seventy six clinical *Nocardia* strains belonging to 12 species were selected according to their high SXT MIC values (≥32:608 μg/ml). These strains were isolated from patients with signs and symptoms of bacterial infection in 40 locations of 27 Spanish provinces between 2007 and 2013. All strains were identified by 16S rRNA partial and full sequencing (Rodriguez-Nava et al., 2006). The sequences were compared with those deposited in the GenBank and leBIBi databases and returned similarities of ≥99.6% with the type species strains (except for five strains that could be identified only at the genus level) according to previously used cut-off for this genus (Petti et al., 2008).

The clinical samples were taken as part of standard patient care and also for this purpose, the bacterial strains were sent to a public national reference laboratory for their identification. This study focused on bacteria and no identifiable human data were used, therefore ethical approval was exempted.

### Antimicrobial Susceptibility Testing

The *Nocardia* cultures were incubated during 48–72 h at 37^∘^C. The colonies were swabbed from blood agar plate, using a sterile swab and transferred to 4.5 ml of sterile water. To each flask, 5-mm sterile glass beads (5–7) were added and repeatedly vortexed, allowing clumps to settle (Clinical Laboratory Standards Institute [CLSI], 2011). The final inoculum was adjusted to a 1.0 McFarland. MICs were determined by *E* test (BioMerieux, Marcy-l’Étoile, France) onto 150 mm MH Blood agar plates, with maximum of five strips for each plate (Glupczynski et al., 2006). The tested agents were: SXT, amoxicillin/clavulanic acid, cefotaxime, imipenem, amikacin, tobramycin, ciprofloxacin, erythromycin, minocycline, sulfonamides, trimethoprim, and linezolid. The MICs were read after 48 h of incubation at 37^∘^C (or after 72 h if growth was weak; Glupczynski et al., 2006). The MIC was defined as the lowest concentration of antimicrobial to inhibit visible growth, except in case of haziness where 80% of inhibition was considered. Resistance was recorded according to CLSI interpretative criteria (Clinical Laboratory Standards Institute [CLSI], 2011); intermediate values were categorized as resistant.

### Detection of Genes for Antibiotic Resistance and Integrases

The presence of antimicrobial resistance genes and class 1–3 integrons was analyzed by PCR in all 76 SXT-resistant *Nocardia* strains. Most of the resistance genes were chosen because of the high frequency with which they appear in clinical and environmental bacteria (given in **Table 1**). On the basis of the *gyrA* sequence of *Nocardia farcinica* IFM 10152 (GenBank accession no. AP006618), primers were used that included the quinolone resistance-determining region (QRDR, 74–113 codons, 5′→3′), i.e., F+61 = CAGCAGGAGATGCAGAACAG, and R-619 = TGTCCAGCGCCCAGTAGAT. PCR products were resolved by electrophoresis on 2% agarose gels. All obtained products were purified using ExoSAP-IT reagent (GE Healthcare, Piscataway, NJ, USA) following the manufacturer’s recommendations, and sequenced by capillary electrophoresis in an ABI Prism 3100 apparatus (Applied Biosystems, Foster City, CA, USA) using the corresponding amplification primers. BLAST software was used to perform identity searches of the GenBank database (http://www.ncbi.nlm.nih.gov).

**Table 1:** Overview of antimicrobial resistance genes and integrons screened and detected (in bold) in the high-level SXT-resistant *Nocardia* strains.

Antibiotic resistance group	Encoded enzymes	Target gene(s) or region	Reference
*Integrase genes*	Class 1 integrase Class 2 integrase Class 3 integrase	***int1 int2 int3***	Su et al. (2006) Su et al. (2006), Ishikawa (2011) Ishikawa (2011)
*Folate pathway inhibitors*			
Sulfonamide resistance	Dihidropteroate synthetases (DHFS)	***sul1 sul2 sul3***	Gosia et al. (2009) Gosia et al. (2009) Byrne-Bailey et al. (2009), Gosia et al. (2009)
Trimethoprim resistance	Dyhidrofolate reductases (DHFR)	***dfrA*(*S1*)*** dfrD, dfrF dfrG, dfrK*	Perreten et al. (2005), Argudín et al. (2011) Dale et al. (1995), Cattoir et al. (2009) Cattoir et al. (2009) Cattoir et al. (2009), Argudín et al. (2011) Argudín et al. (2011), López et al. (2012)
*Betalactam resistance*	Class A betalactamases	***bla_TEM_***** ***blaZ**** bla_SHV_*	Chouchani et al. (2012) Perreten et al. (2005) Henriques et al. (2006a), Azevedo et al. (2013)
	Class B betalactamases^a^	*bla_IMP-1-2-4-7-12_***_,_*bla_V IM_****_-1-_****_2_****_-7_*_,_ *bla_GIM-1_*_,_ *bla_SIM-1_*_,_ *bla_SMP-1_ bla_NDM-1_*	Ellington et al. (2007) Chen et al. (2011)
	Class C betalactamases	*ampC bla_CMY,_*	Bou and Martínez-Beltrán (2000) Frye et al. (2006)
	Class D betalactamases	*oxa-1*, *oxa-2*, *oxa-3*, *oxa-4*, *oxa-7*, *oxa-10*, *oxa-11*, *oxa-13*, *oxa-14*, *oxa-15*, *oxa-16*, *oxa-17*, *oxa-19*, *oxa-21*, *oxa-28*, *oxa-30*, *oxa-31*, *oxa-32*, *oxa-34*, and *oxa-35*	Huovinen et al. (1988), Ouellette et al. (1997), Henriques et al. (2006b)
	Described *Nocardia* betalactamases	*FAR-1 AST-1*	Poirel et al. (2001) Laurent et al. (1999)
*Aminoglycoside resistance*^b^	Aminoglycoside-modifiying enzymes		
	acetyltransferase	*aac*(*6*′)*-Ie-aph*(*2*″)*-Ia,*	Vakulenko et al. (2003)
	phosphotransferases	*aph*(*2*″)*-Ib*, *aph*(*2*″)*-Ic*, *aph*(*2*″)*-Id*	Schmitz et al. (1999)
		*aph*(*3*′)*-IIIa*	Schmitz et al. (1999)
	adenyltransferase	*ant*(*4*″) –Ia, *ant*(*6*′)*-Ia*	Clark et al. (1999), Vakulenko et al. (2003)
	16S rRNA methylases	*rmtA*, *rmtB*, *rmtC*, *rmtD*, and *armA*	Doi and Arakawa (2007)
*Fluoroquinolones*	DNA gyrase mutations	***gyrA***	This study
	Plasmid-mediated quinolone resistance (PMQR) Aminoglycoside acetyl-transferase Eﬄux pump of major facilitator subfamily	*qnrA*, *qnrB*, *qnrC*, *qnrS aac*(*6*′)*-Ib qepA*	Robicsek et al. (2006), Kim et al. (2009) Cattoir et al. (2007) Park et al. (2006) Kim et al. (2009)
*Macrolides*	rRNA adenine N6-methyltransferases	***erm*(*A*), *erm*(*B*),** *erm*(*C*),	Sutcliffe et al. (1996)
	Esterases	*ere*(A), *ere*(B),	Sutcliffe et al. (1996)
	MFS eﬄux proteins	***mef*(*A/E*),** *msrA/B*, ***msrD*,**	Sutcliffe et al. (1996), Malhotra-Kumar et al. (2005), Lüthje and Schwarz (2007)
	Macrolide 2′-phosphotransferases	mph(A)	Sutcliffe et al. (1996)
*Tetracycline*	Ribosomal protection protein genes	***tet*(O), *tet*(M)**	Ng et al. (2001)
	Eﬄux proteins	*tet*(*E*), *tet*(*G*), *tet*(*K*), *and* ***tet*(L)**	Ng et al. (2001)
Linezolid		23S rRNA, *cfr*	Marshall et al. (2002), Kehrenberg and Schwarz (2006)

*^a^Screening only in strains with IMI MIC values ≥ 1mg/L; ^b^gentamicin testing was not included.*

### Nucleotide Sequence Accession Numbers

The new sequences identified for *int1*, *int3*, *sul1*, *sul2*, *dfrA*(*S1*), *blaTEM-1*, *blaZ*, *VIM-2*, *aph*(*3*′)-*IIIa*, *gyrA*, *ermA*, *ermB*, *mefA*, *msrD*, *tet*(O), *tetM*, and *tetL* were deposited in the GenBank database under accession numbers KM194583–KM194606.

## Results

### Distribution and Susceptibilities of SXT-Resistant *Nocardia* Strains

The distribution of *Nocardia* species among the 76 SXT-resistant strains was as follows: *N. abscessus* (*n* = 1), *N. carnea* (*n* = 8), *N. cerradoensis* (*n* = 1), *N. cyriacigeorgica* (*n* = 5), *N. farcinica* (*n* = 24), *N. flavorosea* (*n* = 5), *N. nova* (*n* = 9), *N. otitidiscaviarum* (*n* = 3), *N. rhamnosiphilia* (*n* = 1), *N. shimofusensis* (*n* = 1), *N. transvalensis* (*n* = 8), *N. veterana* (*n* = 5), and *Nocardia* sp. (*n* = 5). These were isolated from 60 respiratory samples (56 sputum, one broncho-aspirate, three broncho-alveolar lavage), six cutaneous abscess, one ulcer, one catheter, six cerebral abscesses, one liver abscess, and one cardiac prosthesis. By species, **Table 2** shows non-susceptibility rates of the highly SXT-resistant *Nocardia* strains (*n* = 76).

**Table 2:** Number and non-susceptibility rates of the highly SXT-resistant *Nocardia* strains (*n* = 76) by species.

Species (no. of strains) Antimicrobials	*Nocardia farcinica* (*n* = 24)	*N. carnea* (*n* = 8)	*N. nova* complex (*n* = 14)^a^	*N. transvalensis* (*n* = 8)	Other species^b^ (*n* = 22)	Total (*n* = 76)
Amoxycillin-clavulanate	4(16.6%)^b^	6 (70.5%)	10 (71.4%)	0	19 (86.3%)	39 (51.3%)
Cefotaxime	9 (37.5)	0	4 (28.5%)	3 (37.5%)	5 (22.7%)	21 (27.6%)
Imipenem	2 (12.0%)	0	1 (7.1%)	3 (37.5%)	6 (27.3%)	12 (15.8%)
Amikacin	0	1 (12.5%)	0	6 (75.0%)	2 (9.1%)	9 (11.8%)
Tobramycin	20 (83.3%)	0	10 (71.4%)	7 (87.5)	4 (18.2%)	41 (53.9%)
Ciprofloxacin	11 (45.8%)	1 (12.5%)	12 (85.7%)	2 (25.0%)	9 (40.9%)	35 (46.0%)
Erythromycin	21 (87.5%)	7 (87.5)	3 (21.4%)	8 (100%)	19 (86.3%)	58 (76.3%)
Minocycline	20 (80.3%)	2 (25.0%)	13 (92.8%)	8 (100%)	14 (63.6%)	57 (75.0%)
Linezolid	2 (8.3%)	0	0	0	1 (4.5%)	3 (3.9%)

*^a^ Include N. nova (n = 9) and N. veterana (n = 5); ^b^N. abscessus (n = 1), N. cerradoensis (n = 1), N. cyriacigeorgica (n = 5), N. flavorosea (n = 5), N. otitidiscaviarum (n = 3), N. rhamnosiphilia (n = 1), N. shimofusensis (n = 1), Nocardia sp. (n = 5). ^b^The following CLSI interpretative criteria for broth microdilution was considered for non-susceptibilities by E test (mg/L): amoxycillin-clavulanate ≥ 16/8, cefotaxime ≥ 16, imipenem ≥ 8, amikacin ≥ 16, tobramycin ≥ 8, ciprofloxacin ≥ 2, minocycline ≥ 2, linezolid ≥ 16. For erythromycin was applied the breakpoint of clarithromycin ≥ 4.*

### Distribution of Integrons and *sul* and *dfr* Genes

Seventy five strains (98.68%) harbored class 1 and/or class 3 integrons (Su et al., 2006; Ishikawa, 2011). Class 1 integrons were more frequently detected than class 3 integrons [93.42% (71/76) vs. 56.57% (43/76)]. The simultaneous presence of class 1 and 3 integrons was seen in 42.10% (32/76) of strains. There was no correlation between species and the absence of class 3 integrons (except in *N. nova*, in which 7/9 strains lacked such integrons). High-level sulfonamide resistance is a consequence of the presence of *sul1–sul3* (plasmid-borne variants of DHPS; Byrne-Bailey et al., 2009; Gosia et al., 2009), and *sul* genes were found in 74 strains (97.36%): 71 strains (93.42%) had *sul1*, and 60 (78.94%) had *sul2.* Both genes were found in 57 strains (75.0%). No class 2 integrons or *sul3* genes were detected in any of the SXT-resistant strains.

Of the 71 strains carrying *sul1*, 69 also carried *intI1*, and 39 also carried *intI3.* Of the 60 strains carrying *sul2*, 56 also carried *intI*, and 34 also carried *intI3*. All the strains were fully resistant to trimethoprim (MICs > 32 mg/L). When screening was performed for the trimethoprim-insensitive *dfr* genes (known to be horizontally transferable in Gram-positive organisms; Dale et al., 1995; Perreten et al., 2005; Cattoir et al., 2009; Argudín et al., 2011; López et al., 2012), i.e., *dfrA*(*S1*), *dfrD*, *dfrF*, *dfrG*, and *dfrK*, *dfrA*(*S1*) was detected in 12 strains belonging to eight species; no other *dfr* gene was detected. The deduced *dfr* proteins for 11 strains were identical to those of *Staphylococcus epidermidis* ATCC 12228 (NP_764674) and *Listeria monocytogenes* (AGU67290). One strain of *N. farcinica* showed a deduced Dfr protein with 24 changes compared to that encoded by *dfrA*(*S1*).

### β-Lactamases and Metallo-β-Lactamases

Two types of β-lactamase gene were detected. The first, *bla*_TEM-1,_ was found in one strain of *N. farcinica* (isolated from a granuloma, with AMC, CTX, and IMP MICs of 32, 4, and 0.5 mg/L, respectively), and in one of *N. nova* (isolated from sputum and with corresponding MICs of 16, 4, and 2 mg/L). The second, *blaZ*, was found in one strain of *N. veterana* (isolated from sputum and with corresponding MICs of 32, 2, and 0.03 mg/L) and in one of *N. flavorosea* (isolated from sputum and with corresponding MICs of 32, 0.15, and 0.012 mg/L).

No other β-lactamase genes, such as *ampC*, *bla*_CMY -2_, and *bla*_OXA_ were found (Bou and Martínez-Beltrán, 2000; Frye et al., 2006; Henriques et al., 2006b). Primers pairs were also designed on the basis of the FAR-1 and AST-1 sequences (GenBank nucleotide nos. AF024601 and AF279904; Laurent et al., 1999; Poirel et al., 2001); but again, no target sequences were detected. Of the metallo-β-lactamases (IMP, VIM, SPM-1, GIM-1, SIM-1, NDM-1; Ellington et al., 2007; Chen et al., 2011) sought, only VIM-2 was detected in one *N. farcinica* strain with no resistance to imipenem (isolated from a cerebral abscess; AMC, CTX, and IMP MICs 1, 4, and 1 mg/L, respectively).

### Aminoglycoside-Modifying Enzymes and 16S rRNA Methylases

Twenty seven amikacin-susceptible and four non-susceptible strains (susceptibility breakpoint ≤8 mg/L) harbored the *aph*(*3*′)*-IIIa* determinant that encodes the 3′-aminoglycoside phosphotransferase [APH(3′)] responsible for amikacin resistance (Schmitz et al., 1999). The strains belonged to different *Nocardia* species, and showed MICs to amikacin ranging from 0.12 to >256 mg/L. No other aminoglycoside-modifying enzyme (AME) genes, i.e., *aac*(*6*′)*-Ie-aph*(*2*″)*-Ia*, *aph*(*2*″)*-Ib*, *aph*(*2*″)*-Ic*, *aph*(*2*″)*-Id*, *ant*(*6*′)*-Ia*, or *ant*(*4*″) –Ia; Clark et al., 1999; Vakulenko et al., 2003); nor 16S rRNA methylases (*rmtA*, *rmtB*, *rmtC*, *rmtD*, and *armA*; Doi and Arakawa, 2007), were detected.

### Plasmid-Mediated Quinolone Resistance, *gyrA*, and other Genes

None of the strains harbored any of the plasmid-mediated quinolone resistance (PMQR) determinants studied (*qnrA*, *qnrB*, *qnrC*, and *qnr*, which encode the pentapeptide repeat proteins that protect type II topoisomerases from quinolone binding; Robicsek et al., 2006; Cattoir et al., 2007; Kim et al., 2009), or the gene for the aminoglycoside acetyltransferase *aac*(*6*′)*-Ib-cr* (which can modify ciprofloxacin; Park et al., 2006), or that for the eﬄux pump *qepA* (Kim et al., 2009).

None of the nucleotide changes observed in *gyrA* would have led to an amino acid sequence with any effect on ciprofloxacin susceptibility or resistance. Within the SXT-resistant *N. carnea*, *N. farcinica*, *N. flavorosea*, *N. nova*, *N. transvalensis*, *N. veterana* species, the amino acid sequences encoded by their *gyrA* gene always appeared to be the same, irrespective of ciprofloxacin resistance status. In *Escherichia coli* it is well known that the mutation from Ser83Ala in *gyrA* leads to change from ciprofloxacin susceptibility to ciprofloxacin resistance. However, in *N. carnea*, *N. farcinica*, *N. flavorosea* and *N. transvalensis*, Ser^83^ appears not to be critical in this respect; all strains of these species possessed Ser^83^ but some were susceptible to ciprofloxacin while others were resistant. In contrast, all the strains of *N. cyriacigeorgica*, *N. nova*, and *N. veterana* showed Ser83Ala, but again, some were susceptible to ciprofloxacin while others were resistant. In all the above *Nocardia* species, no variation was seen in the other position involved in resistance, Asp^87^. Further, in these *Nocardia* species, the *gyrA* positions that affect ciprofloxacin resistance in *Mycobacterium tuberculosis* – Gly^88^ and Asp^94^ – (Maruri et al., 2012) were all exactly the same; all had both Gly^88^ and Asp^94^. Moreover, Ala^90^, another major position affecting ciprofloxacin resistance in *M. tuberculosis*, was also found in the *gyrA* gene of *N. cyriacigeorgica*, *N. nova*, and *N. veterana*, while in *N. carnea*, *N. farcinica*, *N. flavorosea*, and *N. transvalensis* it was changed to Ser^90^.

### Genetic Determinants of Macrolide and Lincosamide Resistance

Screening was performed for genes coding for RNA methylases, i.e., *erm*(A), *erm*(B), *erm*(C), for eﬄux pumps, i.e., *msr*(A)/(B), msr(D), *mef*(A/E), and for inactivating enzymes, i.e., *ere*(A), e*re*(B), *mph*(A) (Sutcliffe et al., 1996; Malhotra-Kumar et al., 2005; Lüthje and Schwarz, 2007). The most common was *erm*(B). This was identified in 59 strains, i.e., in 46 out of 58 strains with erythromycin MICs of ≥4 mg/L, and in 13 out of 18 strains with MICs of ≤2 mg/L. It showed full similarity to the corresponding gene of *Streptococcus pyogenes* (AY357120). The next most common determinant was *mef*(A), which was found in 11 strains [three *N. carnea* strains (with erythromycin MICs of 4, 8, and 16 mg/L), five *N. farcinica* strains (2, 8, 16, 32, and 32 mg/L), one *N. flavorosea* strains (2 mg/L), one *N. transvalensis* strain (4 mg/L), and one *Nocardia sp.* (1 mg/L)]. This in turn was followed by *msr*(D) in four strains: one *N. farcinica*, one *N. flavorosea*, and two *N. transvalensis* strains with erythromycin MICs of 32, 32, 16, and 4 mg/L, respectively. The next most common was *erm*(A) subclass *erm*TR, which was found in two strains (one strain each of *N. farcinica* and *N. transvalensis* (erythromycin MICs 32 and 64 mg/L).

The resistance genotypes found in combination with *erm*(B) were: *erm*(B) alone in 51 strains, *erm*(B) + *erm*(A) in one strain, *erm*(B) + *mef*(A) in one strain, *erm*(B) + *msr*(D) in two strains, *erm*(B) + *erm*(A) + *mef*(A) in one strain, *erm*(B) + *msr*(D) + *mef*(A) in two strains, and *mef*(A) alone in seven strains. The *erm*(A) gene in the studied *Nocardia* species was identical to those expressed in *S. pyogenes*, *S. pneumoniae*, and *Aerococcus urinae* (CP000262, CP002121, and CP002512). The macrolide-eﬄux gene *mef*(A) and its protein were the same as those seen in *S. mitis* (DQ304773) but with one amino acid change encoded compared to *S. pyogenes*, *S. pneumoniae*, and *S. suis* (CP000003, emb FQ312029, CP003922). The macrolide-specific ABC-type eﬄux carrier gene *msr*(D) also coded for one change at the amino acid level with respect to the corresponding genes in *S. pyogenes*, *S. pneumoniae*, and *S. suis* (CP000003, emb FQ312029, CP002465). This involved the replacement of Glu^224^ (GAA) in *msrD* of *S. pyogenes* MGAS10394 (CP000003) by Gly^224^ (GGA). This erythromycin eﬄux resistance gene has been described for *N. seriolae* isolated from yellowtail fish (AB518863-5), but coding for one amino acid difference. None of the *Nocardia* under study harbored *erm*(C), *msr*(A)/(B), *ere*(A), *ere*(B), or *mph*(A).

Two *N. farcinica* strains and one *N. cyriacigeorgica* strain showing linezolid resistance by *E* test (MICs of 64, >256, and >256 mg/L, respectively) and microdilution, did not show the mutations in domain V of the 23S RNA (G2576T or T2504A) described for different *Staphylococcus* and *Enterococcus* species (Marshall et al., 2002). Nor did they harbor the *cfr* gene, which has been reported in some resistant strains of the latter bacteria.

### Genetic Determinants of Tetracycline Resistance

Among the 57 minocycline-resistant strains (MIC > 1 mg/L), the ribosomal protection protein gene *tet*(*O*) was the most common determinant, followed by *tetM*, and then a long way behind by the eﬄux pump gene *tetL* (in 27, 13, and 2 strains, respectively). Nineteen SXT-resistant, minocycline-susceptible strains were positive for *tet*(O), *tet*(M), and *tet*(L) (10, 6, and 1 strains). The *tet*(O) and *tet*(M) genes coded for an amino acid sequence identical to those deduced for *S. pyogenes* MGAS2096 and SP94 (CP000261 and JQ001862), while *tet*(L) coded for a sequence identical to that reported for *N. seriolae*, *S. suis* (AB513330, JQ280448) and *Bacillus* species. The genotypes (and number of strains) observed were: *tet*(O) (25), tet(M) (7), *tet*(L) (2) *tet*(O) + *tet*(M) (11), and *tet*(O) + *tet*(M) + *tet*(L) (1). *tet*(E), *tet*(G), and *tet*(K) (Ng et al., 2001) were not detected.

## Discussion

*Nocardia* are environmental bacteria that live in soil, water, rotting vegetation, and other organic matter. However, they can cause severe infections in the human respiratory tract, skin, and subcutaneous tissues; on some occasions they may even infect the central nervous system (Brown-Elliott et al., 2006; Minero et al., 2009; Ambrosioni et al., 2010). Like other actinobacteria, they recycle organic material and produce a wide range of biological compounds, including antibiotics (Waksman et al., 2010). In the environmental setting, the presence of antimicrobials can lead to exposed bacteria becoming resistant (Cantón, 2009). Such resistance may involve changes in the permeability of the cell wall to the antimicrobial agent, the loss of its main target, or the increased expression of eﬄux pumps or inactivating enzymes. Resistance traits can be acquired via mutations in pre-existing chromosomal genes, or via the capture of mobile resistance determinants through horizontal gene transfer (Cantón, 2009; Djordjevic et al., 2013). It is known that resistance genes can move between different parts of the microbial biosphere. Environmental bacteria participate in this by acting as conduits for the spread of antibiotic resistant genes (Martínez, 2008; Roberts, 2011; Stalder et al., 2012; Walsh, 2013). Multidrug resistance is widely considered to be driven via the pressure exerted by the use of antimicrobials. These agents are commonly used in the hospital and community (Woodford et al., 2014). One might expect that environmental bacteria such as *Nocardia*, with niches in soil, water, and vegetation but which do not make up part of the normal human flora, might be affected by antimicrobials in a different way. However, the present results show that resistance determinants are commonly found in strains of *Nocardia* sp. causing opportunistic infection in humans.

This work examines a diverse group of *Nocardia* strains strongly resistant to SXT, the standard treatment for localized and disseminated nocardiosis. Their overall susceptibility profiles were studied by the *E* test, since difficulties (deficient growth in the microdilution wells, problems reading the endpoints, etc.) are often encountered with the current CLSI recommendation for susceptibility testing of *Nocardia,* the broth microdilution method. By *E* test, not yet approved by CLSI, the results are easily evaluated, and a wide range of dilutions of antimicrobials can be examined simultaneously. In *Nocardia*, susceptibility phenotypes are strongly species-dependent (Schlaberg et al., 2014). Thus, to avoid any bias caused by the species composition of the SXT-resistant group, MICs data were provided separately.

Resistance to SXT may compromise its continued use as a treatment of nocardiosis. In the present work, resistance to sulfonamide and trimethoprim can be partially explained by the presence of *dfr* genes which encode insensitive DHPS and DFR enzymes, and even better explained by the presence of integrons carrying *sul* genes. Nearly all of the SXT-resistant strains carried *sul1* as one of the backbone genes of the 3′-conserved segments in class 1 integrons, and three quarters carried *sul2* (probably encoded by a small non-conjugative plasmid or a medium–large plasmid), as seen in other genera (Hu et al., 2011). Of the trimethoprim resistance genes reported in Gram-positive bacteria, *dfrA*(S1), dfr(D), *dfr*(*F*), *dfr*(*G*), and *dfr*(*K*) (Perreten et al., 2005; Cattoir et al., 2009), only *dfrA*(S1) was seen in the present SXT-resistant *Nocardia* (14.4%). The *dfrA*(S1) gene is commonly associated with class 1 integrons. Another mechanism is required to explain the strong trimethoprim resistance shown by the other 64 strains.

The clinically important class 1 integrons, which have the potential to allow for the co-selection of antibiotic resistance, are widely disseminated in environmental settings, associated with plasmids of the IncP-1 incompatibility group. These integrons, and these plasmids, are commonly detected in bacteria living in estuarine waters, wastewater treatment plants, agricultural soils, and the rhizosphere (Henriques et al., 2006a; Stalder et al., 2012). Class 2 integrons are described as the second most common, but none was detected in the present work. However, nearly half of the studied strains carried class 3 integrons – which have been little reported in the literature. Of the five types of class 3 integrons described to date, three were detected in clinical enterobacteria (Arakawa et al., 1995; Correia et al., 2003; Poirel et al., 2010; Barraud et al., 2013). Those detected in the present work in *Nocardia* were identical to those of the environmental bacteria *Delftia tsuruhatensis* and *D. acidovorans* (GenBank accession no. EF469602–EF467661), and correspond to the Inc3-4 type (Xu et al., 2007). To date, the presence of class 3 integrons in the clinical setting has been anecdotal, but in environmental ecosystems they might play a role as an important pool of resistance determinants (Stalder et al., 2012).

Two class A β-lactamases – FAR-1 and AST-1 – were described *in N. farcinica* and *N. asteroides sensu* stricto (Laurent et al., 1999; Poirel et al., 2001), but they cannot explain the complete β-lactam resistance phenotype. This is the first time that *bla*_TEM-1_ and *bla*Z, with a ubiquitous distribution in Gram-positive and Gram-negative bacteria, have been reported for *Nocardia* strains (*n* = 4). One of the so-called “big five” carbapenemases, VIM-2, was curiously found in an imipenem-susceptible *Nocardia* strain. Most of the carbapenemases acquired by bacterial species of clinical importance are thought to have had their origin in environmental bacteria. However, the environment might also be contaminated by resistant bacteria of clinical origin (Woodford et al., 2014). The environmental sources of the bacteria carrying metallo-carbapenemase genes include rivers, other water, and sewage, all of which are also common habitats of *Nocardia* species. Monitoring carbapenemases in environmental bacteria might help us determine how widely they become disseminated.

High-level aminoglycoside resistance is mediated by AMEs. Curiously, the only AME gene found in the present *Nocardia* strains (*n* = 31) was *aph*(3′)-IIIa, which inactivates kanamycin and amikacin. It has also been described in *Bacillus anthracis*, *Clostridium perfringens*, *Enterococcus faecalis*, *Streptococcus*, and *Staphylococcus* (Perreten et al., 2005; Zarrilli et al., 2005). However, this very same determinant was also present in the amikacin-susceptible strains of *Nocardia*, and has even been reported in some *E. faecalis* strains with low to intermediate amikacin resistance (Zarrilli et al., 2005). In the aminoglycoside-producing actinomycetes, 16S rRNA methylases have been detected that are said to protect the 16S RNA in the 30S ribosome subunit against aminoglycoside (Doi and Arakawa, 2007). None of the *Nocardia* strains studied in the present work carried any of these genes.

High-level resistance to fluorquinolone in Gram-negative bacteria is produced by mutations in *gyrA*, the most common mutation site being Ser^83^, which can change to code for Leu, Trp, Phe, or Tyr. However, in *M. tuberculosis*, which like *Nocardia* is an actinomycete, fluorquinolone resistance mainly occurs via modifications at positions 88, 90, or 94 in the QRDR of *gyrA* (Maruri et al., 2012). In the present *Nocardia* strains, no association was seen between high-level resistance/non-susceptibility to fluorquinolone and the distribution of mutations in these hot spots. Among the studied *Nocardia* species, not only the amino acid differences in positions 83 (Ser or Ala) and 90 (Ala or Ser), but also other 20 amino acids of positions 36, 48, 53, 58, 71, 72, 84, 97, 98, 103, 105, 134,141, 146, 147, 151, 152, 153, 154, and 188 (respect *E. coli* numbering) varied. These GyrA changes were not related to ciprofloxacin susceptibility, but yes to species polymorphism.

Macrolides and lincosamides inhibit the synthesis of protein by binding to the 50S ribosomal unit, and therefore block peptide bond formation and/or translation. The dimethylation of the adenine residue in the 23S rRNA produces a conformational change in the ribosome leading to a constitutive or inducible macrolide – but not linezolid – resistance phenotype (Roberts, 2011). The most common bacterial rRNA methylase is ErmB, which confers high MICs for macrolides; this was seen in the present work for 77.6% of all the *Nocardia* strains examined. *erm*(B) is carried on either conjugative transposons, such as those of the Tn*916* family (in chromosome or plasmids), or in non-conjugative transposons such as Tn*917*. It is well known that Tn*916* and its relatives (Tn*1545*, Tn*3703*, Tn*3704*, Tn*3872*, Tn*6002*, and Tn*6003*), as well as newly found elements, often carry other resistance determinants, including *tet*(M), *tet*(O), and *mef*(A)-*msr*(D), among others (Brenciani et al., 2007; Cochetti et al., 2008; Roberts, 2011).

The second most commonly detected resistance mechanism in the present *Nocardia* was related to the possession of *mef*(A), which was detected in some 14.4% of the studied strains. Both *mef*(A) and *mef*(E) code for an active macrolide eﬄux pump mediated by the ABC transporter, the transmembrane domains of which are encoded by the *mef*A/E genes, and the ATP-binding domains by *msr*(D) (Malhotra-Kumar et al., 2005). *mef*(A) and *msr*(D) are always linked in other Gram-positive bacteria (Roberts, 2011), but, in the present work, two of the four *msr*(D)-positive *Nocardia* strains detected showed no such combination. Regarding the three linezolid-resistant *Nocardia* strains, the described mutations of 23S (G2576T or T2504) or the *cfr* gene were no detected (Marshall et al., 2002).

The same Tn*916* family mobile elements that carry the major macrolide resistance determinants also carry tetracycline determinants (Roberts, 2005, 2011), as seen in *S. pyogenes*, in which the strongly linked *erm*(B) and *tet*(M) genes are carried on highly variable and complex genetic elements (Brenciani et al., 2004, 2007; Cochetti et al., 2008; Roberts, 2011). However, the association between these determinants was quite different in *Nocardia*. The combination *erm*(B) + *tet*(O) appeared nearly twice as often as *erm*(B) + *tet*(M) (33 strains compared to 18). *erm*(B) + *tet*(O) + *tet*(M) appeared in 12 strains.

The presence of several resistance determinants – *VIM*-2, *aph*(*3*′)*-IIIa*, *erm*(B), *mef*(A), *tet*(O), *tet*(M) – in strains susceptible to the involved antimicrobials, may mean that, in these strains, these determinants are silent genes or may lack functionality. In environmental bacteria, the detection of a specific resistant determinant with susceptible phenotype, could be suggested a different role of the resistance mechanism to the “weapon-shield” (Martínez, 2008), or some failure or difference in their acquisition that affect their expression.

The clinical SXT-resistant *Nocardia* strains analyzed here were very similar with regard to their antimicrobial resistance traits, perhaps the result of a common genetic platform. Although sulfonamides are synthetic compounds and their use in human infections has been reduced for decades, sulfonamide resistance determinants have been shown to persist in bacteria isolated from humans, animals, food, and the environment. sul-carrying plasmids form a pool of resistance genes that can be transferred to human and non-human reservoirs, as observed in this work for the SXT-resistant *Nocardia* strains. These findings are of some concern.

## Conclusion

This is the first work to describe in *Nocardia* the presence of several genetic determinants frequently involved in antimicrobial resistance in clinical bacteria. The extent of the environmental resistome, and the recruitment of multiple resistance genes from it, should be further studied.

## Conflict of Interest Statement

The authors declare that the research was conducted in the absence of any commercial or financial relationships that could be construed as a potential conflict of interest.
